# Tissue Biomarkers Outperform Plasma Signals in Pelvic Organ Prolapse: Evidence of a Dominant Extracellular Matrix Remodelling Phenotype

**DOI:** 10.3390/cimb48090952

**Published:** 2026-09-18

**Authors:** Bojan Vučković, Ivan Ignjatović, Milan Potić, Slavica Stojnev, Miloš Dičić, Dušan Sokolović

**Affiliations:** 1 Urology Department, General Hospital Prokuplje, 18400 Prokuplje, Serbia; drbojanvuckovic@gmail.com; 2 Faculty of Medicine, University of Niš, 18000 Nis, Serbia; ignjatovic1964@gmail.com (I.I.); uropota@gmail.com (M.P.); slavicastojnev@gmail.com (S.S.); 3 Clinic for Urology, University Clinical Center Nis, 18000 Nis, Serbia; 4 Center for Pathology and Pathological Anatomy, University Clinical Center Nis, 18000 Nis, Serbia; 5 Department of Histology, Faculty of Medicine, University of Niš, 18000 Nis, Serbia; dicic@hotmail.com; 6 Department of Biochemistry, Faculty of Medicine, University of Niš, 18000 Nis, Serbia; 7 Department of Laboratory Diagnostics, Institute for Treatment and Rehabilitation “Niška Banja”, 18205 Niska Banja, Serbia

**Keywords:** pelvic organ prolapse, extracellular matrix, MMP-2, MMP-9, tissue remodelling

## Abstract

Pelvic floor disorders, particularly pelvic organ prolapse (POP) with or without stress urinary incontinence (SUI), are among the most prevalent benign conditions affecting women and are associated with substantial burdens. This study aimed to evaluate tissue extracellular matrix remodelling in women with POP with or without SUI by integrating clinical and phenotypic characteristics, and tissue and plasma specimens, and to determine whether the dominant biological signal is observed at the systemic or local tissue level in pelvic floor failure. Case–control analysis consisted of 121 women with POP with or without SUI and a control group (POP and SUI-free). Plasma and tissue concentrations of collagen type I, collagen type III, elastin, MMP-1, MMP-2, MMP-3, and MMP-9 were quantified (60 POP with or without SUI, 61 controls). Clinical and demographic characteristics were compared between groups, and secondary correlations between tissue biomarkers, pelvic floor symptom scores, and POP-Q parameters were evaluated within the POP group. The results of the present study reveal that the studied women were older, had higher BMI, parity, and vaginal delivery burden, and had markedly higher symptom scores. Plasma biomarkers showed limited differences between groups, whereas tissue biomarkers showed strong separation: COL1 and ELN were lower, while COL3, MMP1, MMP2, MMP3, and MMP9 were higher in POP. The strongest individual discriminatory markers were found to be MMP2 (AUC 0.958) and MMP9 (AUC 0.977). In adjusted models controlling age, BMI, and parity, parameters such as COL1, COL3, ELN, MMP2, MMP3, and MMP9 remained independently associated with POP. In this cohort, pelvic organ prolapse is associated with a robust extracellular matrix remodelling signature in vaginal-wall tissue, characterised by reduced structural components and increased MMP levels. Tissue biomarkers, particularly MMP-2 and MMP-9, may represent markers of the POP-associated tissue phenotype and warrant further validation in larger cohorts.

## 1. Introduction

Pelvic floor disorders, particularly pelvic organ prolapse (POP) and stress urinary incontinence (SUI), are among the most prevalent benign conditions affecting women. These disorders are associated with substantial symptom burden, impaired quality of life, sexual dysfunction, and an increased lifetime risk for surgical intervention [1,2]. Established epidemiological and clinical studies have consistently linked POP and SUI to parity, vaginal delivery, ageing, obesity, and other chronic loading factors, yet these risk factors alone do not fully explain the reason why only a subset of exposed women develop clinically significant pelvic floor failure [1,2,3,4]. Current concepts, therefore, view POP and SUI not simply as passive consequences of ageing or childbirth, but as the result of an interaction among mechanical loading, tissue quality, pelvic floor architecture, and biological susceptibility [4,5].

The extracellular matrix (ECM) is a central part of the biological background of this disorder. Pelvic support depends on the integrity of collagen-rich connective tissue, elastic fibres, and matrix-regulating enzymes that preserve the mechanical competence of the vaginal wall and supporting ligaments [4,5,6]. Collagen type I is the principal load-bearing collagen and confers tensile strength, whereas collagen type III is more closely related to compliance and repair. Elastin contributes to recoil and resilience, allowing tissues to recover after repetitive deformation [6,7,8]. The disturbance of this balance has repeatedly been implicated in POP. Classical biochemical and histological studies demonstrated altered collagen turnover, increased collagen remodelling, and elastin abnormalities in prolapsed tissues [6,7,8]. More recently, a systematic review and meta-analysis published confirmed that women with POP exhibit a reproducible pattern of extracellular matrix dysregulation, including lower collagen I and elastin expression together with higher collagen III and higher expression of several matrix metalloproteinases (MMPs), particularly MMP-1, MMP-2, and MMP-9 [9].

These changes are plausible because MMPs, together with their tissue inhibitors, are considered major regulators of ECM turnover. Excess proteolytic activity can promote collagen fragmentation, elastin degradation, and progressive weakening of supportive tissues. In parallel, recent reviews and molecular studies have emphasised that POP susceptibility also reflects broader disturbances in connective tissue homeostasis, hormone-related signalling, oxidative stress, and mechanosensitive pathways such as integrin/TGF-beta signalling [10,11,12]. Recent transcriptomic and bioinformatic analyses have further reinforced this concept by identifying dysregulated RNA networks, focal adhesion pathways, ECM pathways, and candidate therapeutic targets in prolapsed uterosacral ligament and vaginal tissues [13,14,15]. Collectively, these data support the view that the dominant disease phenotype is expressed locally at the tissue level, where structural matrix depletion and increased proteolysis converge.

The present study examines whether surgically treated POP, with or without SUI and related pelvic floor dysfunction, are primarily associated with a localised ECM-remodelling phenotype, aiming to strengthen and give clearer insight into the pathophysiological interpretation of tissue biomarkers in prolapse. This was to be done by integrating clinical characteristics of surgically treated women, with POP and a corresponding control group, with plasma and tissue biomarkers reflecting tissue remodelling.

## 2. Materials and Methods

### 2.1. Study Design

A single-centre, observational case–control study was conducted at the Department of Urology, Health Centre Prokuplje, Serbia, during 2025 (a 12-month period). The study included women who underwent surgical treatment for POP with or without SUI, as well as a control group of women undergoing gynaecological surgery for benign non-prolapse indications. The study was conducted in accordance with the Declaration of Helsinki and approved by the Ethics Committee of the Health Centre Prokuplje (approval No. 7186, 10 October 2024) and the Ethics Committee of the Faculty of Medicine, University of Niš (approval No. 12-16442-1/2-2, 20 December 2024). Written informed consent was obtained from all participants prior to their involvement in the study. All procedures were also performed in accordance with institutional clinical protocols.

### 2.2. Study Population

A total of 121 women were included in the analysis and allocated into two groups: the case group (POP/SUI group) consisted of 60 women who underwent surgical treatment for POP, with or without stress urinary incontinence SUI. The cohort was dominated by POP, with SUI present in a subset of patients. No isolated SUI cases were present, and all SUI occurred in combination with prolapse. The case group included 45 patients with isolated POP and 15 with combined POP + SUI. All patients had clinically confirmed pelvic floor dysfunction requiring surgical intervention. POP was assessed using the standardised Pelvic Organ Prolapse Quantification (POP-Q) system. Inclusion criteria for the POP/SUI group were participants who met all of the following criteria: age ≥ 18 years, clinically confirmed POP with or without SUI, indication for surgical treatment, POP-Q stage I or higher (for prolapse cases), availability of adequate intraoperative tissue sample, and a complete clinical dataset including key demographic and pelvic floor variables.

The control group included 61 women without clinically significant POP or SUI, with or without SUI, who underwent surgery for benign, non-prolapse gynaecological indications. These indications included: uterine leiomyoma, abnormal uterine bleeding, adenomyosis, and other benign conditions requiring surgical treatment. Controls had no symptoms suggestive of prolapse (no vaginal bulge or pressure), without indication for pelvic floor reconstructive surgery and no clinically significant pelvic organ prolapse on examination. Participants were included in the control group if all of the following criteria were met: age ≥ 18 years, undergoing surgery for a benign non-prolapse indication, absence of clinically significant POP, POP-Q stage 0 or I, absence of prolapse-related symptoms, no SUI, no indication for pelvic floor reconstructive surgery, availability of an adequate tissue sample from the same anatomical site, and a complete clinical dataset.

Participants were excluded from the study if any of the following were present: malignant pelvic disease, prior pelvic radiotherapy, active pelvic or systemic infection, pregnancy, systemic connective tissue disorders, chronic inflammatory or autoimmune diseases, long-term corticosteroid or immunosuppressive therapy, inadequate or non-standardised tissue sample, or incomplete clinical or laboratory data.

### 2.3. Clinical and Phenotypic Assessment

For all participants, clinical and demographic variables were recorded throughout: age, body mass index (BMI), parity, number of vaginal deliveries, Caesarean section history, and duration of symptoms. Pelvic floor status was assessed using the POP-Q system, and symptom assessment utilised validated questionnaires: UDI-6 (Urogenital Distress Inventory-6) and PFDI-20 (Pelvic Floor Distress Inventory-20).

### 2.4. Plasma Biomarker Analysis

Venous blood samples were collected preoperatively into EDTA-containing tubes. Samples were centrifuged at 3000 rpm for 10 min to separate plasma. The plasma was aliquoted and stored at −80 °C until analysis. Plasma concentrations of collagen type I (COL1), collagen type III (COL3), elastin (ELN), matrix metalloproteinase-1 (MMP-1), MMP-2, MMP-3, and MMP-9 were quantified using commercially available enzyme-linked immunosorbent assay (ELISA) kits according to the manufacturers’ instructions (Fine Test, Wuhan, China). According to the manufacturer, the assay ranges and lower limits of detection (sensitivity) were 0.313–20 ng/mL and <0.188 ng/mL for COL1 (EH0958), 31.25–2000 pg/mL and <18.75 pg/mL for COL3 (EH0746), 0.469–30 ng/mL and <0.281 ng/mL for ELN (EH1505), 78.125–5000 pg/mL and <46.875 pg/mL for MMP-1 (EH0232), 0.5–32 ng/mL and <0.191 ng/mL for MMP-2 (EH0017), 0.156–10 ng/mL and <0.094 ng/mL for MMP-3 (EH0235), and 0.313–20 ng/mL and <0.188 ng/mL for MMP-9 (EH0238). All measurements were performed in duplicate, and mean values were used for analysis. Biomarker concentrations were expressed in ng/mL.

### 2.5. Tissue Sampling and Biomarker Analysis

Tissue samples were obtained intraoperatively from a predefined and standardised anatomical site, as a 5 × 5 mm full-thickness part of the anterior vaginal wall, 30 mm lateral to the cervix on the left side. To minimise sampling bias, tissue in both groups was obtained from the same anatomical location using a standardised surgical protocol and processed under identical laboratory conditions.

For tissue analysis, samples of the anterior vaginal wall were obtained intraoperatively and immediately processed. Tissue specimens were rinsed in cold phosphate-buffered saline (PBS), weighed, and mechanically homogenised under standardised conditions. Homogenates were centrifuged at 10,000 rpm for 15 min at 4 °C, and the supernatants were collected for analysis.

Tissue concentrations of COL1, COL3, ELN, MMP-1, MMP-2, MMP-3, and MMP-9 were quantified using ELISA assays adapted for tissue homogenates following the same standardised protocols (Fine Test, Wuhan, China). The manufacturer lists cell/tissue lysate among applicable sample types and provides a general tissue-homogenate preparation protocol; however, the available manufacturer validation is not specific to vaginal-wall tissue. No study-specific matrix validation in vaginal tissue (such as spike-recovery, dilutional parallelism, or matrix-interference testing) was performed. Potential vaginal-tissue matrix effects therefore cannot be excluded. Results were normalised to the pre-homogenisation wet tissue weight measured after PBS rinsing and immediately before homogenisation and were expressed as ng/mg of wet tissue; total soluble protein concentration was not used as the normalisation denominator. Because wet-weight normalisation may be influenced by between-sample variation in tissue hydration, this potential source of pre-analytical variability was considered when interpreting tissue biomarker concentrations. To minimise inter-assay variability, all samples from the POP/SUI group and the control group were analysed in the same experimental batches under identical laboratory conditions. Collected tissue samples were immediately processed and analysed for extracellular matrix composition.

### 2.6. Histopathological Analysis

Part of the collected tissue samples from both the POP/SUI and control groups were immersed in 10% neutral-buffered formalin fixative and further processed. Following fixation, the samples were processed through graded alcohols for dehydration and embedded in paraffin wax. Paraffin blocks were sectioned at 4–5 μm thickness and prepared for staining according to a routine histochemical staining protocol; hematoxylin and eosin (H&E), Van Gieson (VG) and Masson’s Trichrome (MT). Histopathological examination was performed using light microscopy, and representative photomicrographs were obtained.

Vimentin expression was evaluated by immunohistochemistry on formalin-fixed, paraffin-embedded tissue sections. Briefly, 4–5 μm thick sections were deparaffinized in xylene, rehydrated through graded alcohols, and rinsed in distilled water. Antigen retrieval was performed by heating the sections in citrate buffer (pH 6.0). Endogenous peroxidase activity was blocked using 3% hydrogen peroxide, followed by incubation with a protein blocking solution to reduce nonspecific binding. Sections were then incubated with a primary anti-vimentin antibody at the recommended dilution (Merck, Darmstadt, Germany), followed by an appropriate secondary antibody and detection using a horseradish peroxidase/DAB visualisation system. The sections were counterstained with hematoxylin, dehydrated, cleared, and mounted. Positive vimentin immunoreactivity was identified as brown cytoplasmic staining and evaluated by light microscopy.

### 2.7. Statistical Analysis

Statistical analysis was performed using IBM SPSS Statistics v27.0 (IBM Corp., Armonk, NY, USA); resampling-based internal validation was additionally performed in Python 3.13.5 using scikit-learn 1.8.0. Continuous variables were expressed as mean ± standard deviation or median (interquartile range), according to distribution, and categorical variables as counts and percentages. Normality was assessed using the Kolmogorov–Smirnov test. Between-group comparisons used Student’s *t*-test for approximately normally distributed variables and the Mann–Whitney U test otherwise. Effect sizes were reported as Cohen’s d for Student’s *t*-test and Cliff’s delta (δ) for Mann–Whitney comparisons. Multivariable logistic regression models were used to evaluate associations between individual tissue biomarkers and POP/SUI status after adjustment for age, BMI, and parity; results are presented as odds ratios with 95% confidence intervals. Receiver operating characteristic (ROC) analysis was used to assess discrimination. Four candidate panels were evaluated: MMP2 + MMP9 tissue, COL3 + MMP2 + MMP9 tissue, COL1 + COL3 + ELN + MMP2 + MMP9 tissue, and ELN + COL1 + MMP3 plasma. The candidate marker sets were selected after examination of the full-cohort univariate and adjusted results, based on biological plausibility and discriminatory performance, and were then fixed before resampling. No additional data-driven feature selection was performed within cross-validation. Internal validation used stratified 10-fold cross-validation with shuffling and a fixed random seed of 42, with one complete 10-fold repetition. Within each training fold, z-score standardisation, logistic-regression model fitting, and determination of the classification threshold by the Youden index were performed exclusively using training-fold data; the held-out fold was used only for prediction and performance assessment. Fold-specific AUCs were summarised as mean ± standard deviation, and pooled out-of-fold probabilities were used to calculate an overall cross-validated AUC. Sensitivity, specificity, positive predictive value (PPV), and negative predictive value (NPV) were calculated from held-out classifications obtained using the corresponding training-fold threshold. Ninety-five percent confidence intervals for pooled out-of-fold AUC, sensitivity, specificity, PPV, and NPV were estimated using percentile bootstrap resampling of the held-out observations with 5000 resamples. Spearman correlation coefficients were used to evaluate relationships between tissue biomarkers, symptom scores, and POP-Q parameters. To assess associations with clinical severity independently of case–control separation, the correlations were recalculated within the POP/SUI group only (*n* = 60). All tests were two-sided, with *p* < 0.05 considered statistically significant.

## 3. Results

### 3.1. Study Population Description

The present study included 121 women, 60 in the POP/SUI group and 61 in the control group. Patients from the POP/SUI group were significantly older than controls and had significantly higher BMI (Table 1). Parity and number of vaginal deliveries were also significantly higher in the POP/SUI group (Table 1). Caesarean section history did not differ between groups (*p* = 0.988). Symptom burden was markedly higher in the POP/SUI group for both UDI-6 and PFDI-20 scores (Table 1). Within the 60 surgically treated POP cases, POP-Q stage was re-derived from the recorded POP-Q point measurements according to standard POP-Q staging criteria: stage II in three women (5.0%), stage III in 55 (91.7%), and stage IV in two (3.3%); no stage I surgical cases were present. Because the stage II and stage IV subgroups were small, no formal stage-stratified biomarker analysis was performed.

### 3.2. Plasma Biomarker Changes

Significant differences were observed for COL1 plasma (10.27 vs. 13.81, *p* = 0.007) and ELN plasma (2.49 vs. 3.20, *p* < 0.001) levels between the POP/SUI and the control group (Table 2). The remaining studied plasma biomarkers were not statistically significant (Table 2).

### 3.3. Tissue Biomarker Changes

**Figure 1:**
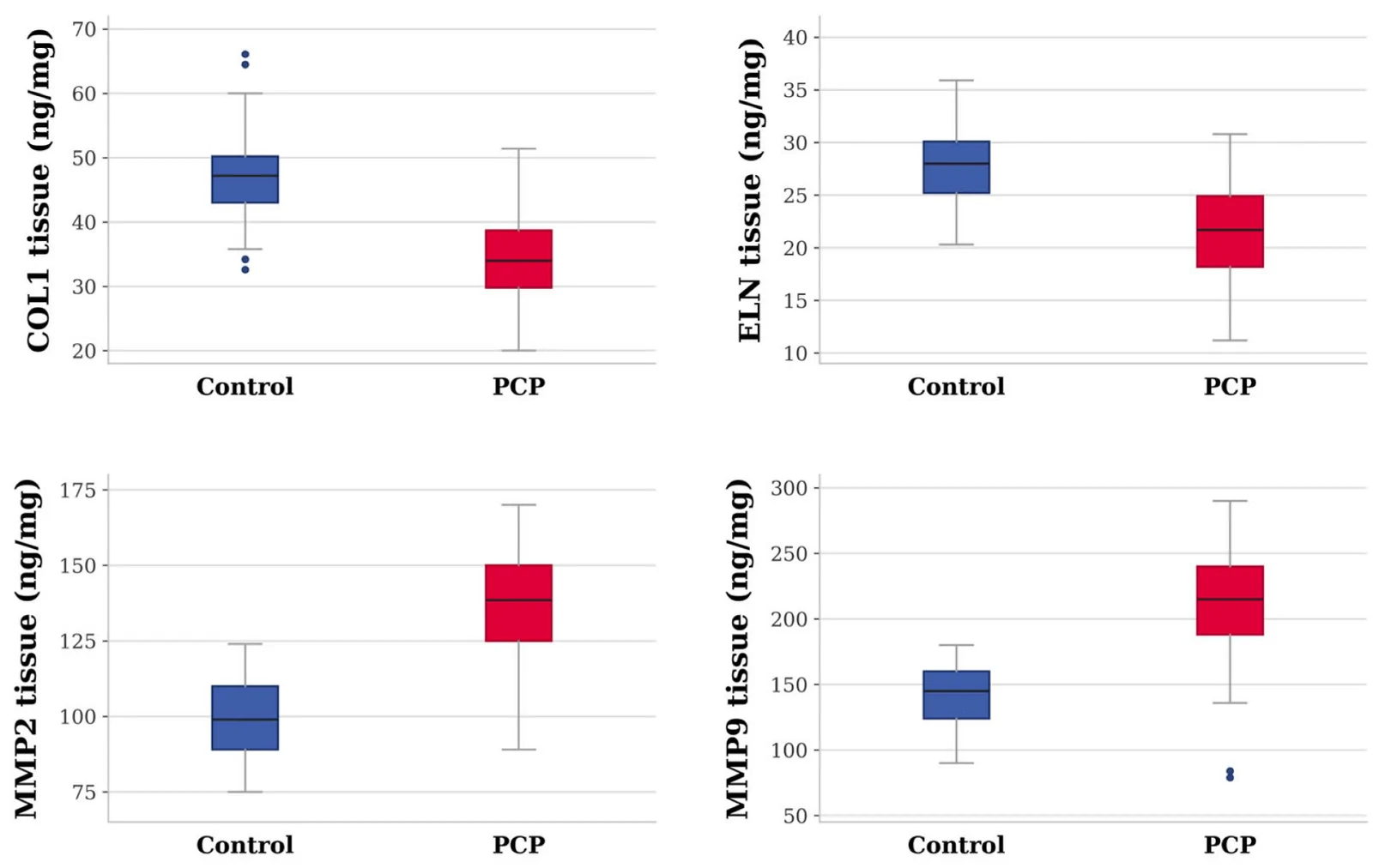
Distribution of tissue ECM biomarkers. In the POP/SUI group of patients, decreased COL1 and ELN and increased COL3, MMP2, and MMP9 were observed.

Tissue levels of COL1 and ELN were significantly lower in the POP/SUI group when compared to the control (Table 3, Figure 1). On the other hand, COL3 tissue levels were higher in the POP/SUI group (41.11 ± 6.00 vs. 33.23 ± 5.78 ng/mg, *p* < 0.001, AUC 0.818). All four studied metalloproteinases, MMP1, MMP2, MMP3, and MMP9, were found to be significantly higher in the POP/SUI group. The strongest separations were seen for MMP2 (132.44 vs. 98.72, *p* < 0.001, AUC 0.958) and MMP9 (216.63 vs. 145.02, *p* < 0.001, AUC 0.977). Every tissue marker remained significant in univariate testing, indicating a broad extracellular matrix remodelling signature rather than an isolated single-marker effect.

### 3.4. Adjusted Tissue Models and Discrimination

**Figure 2:**
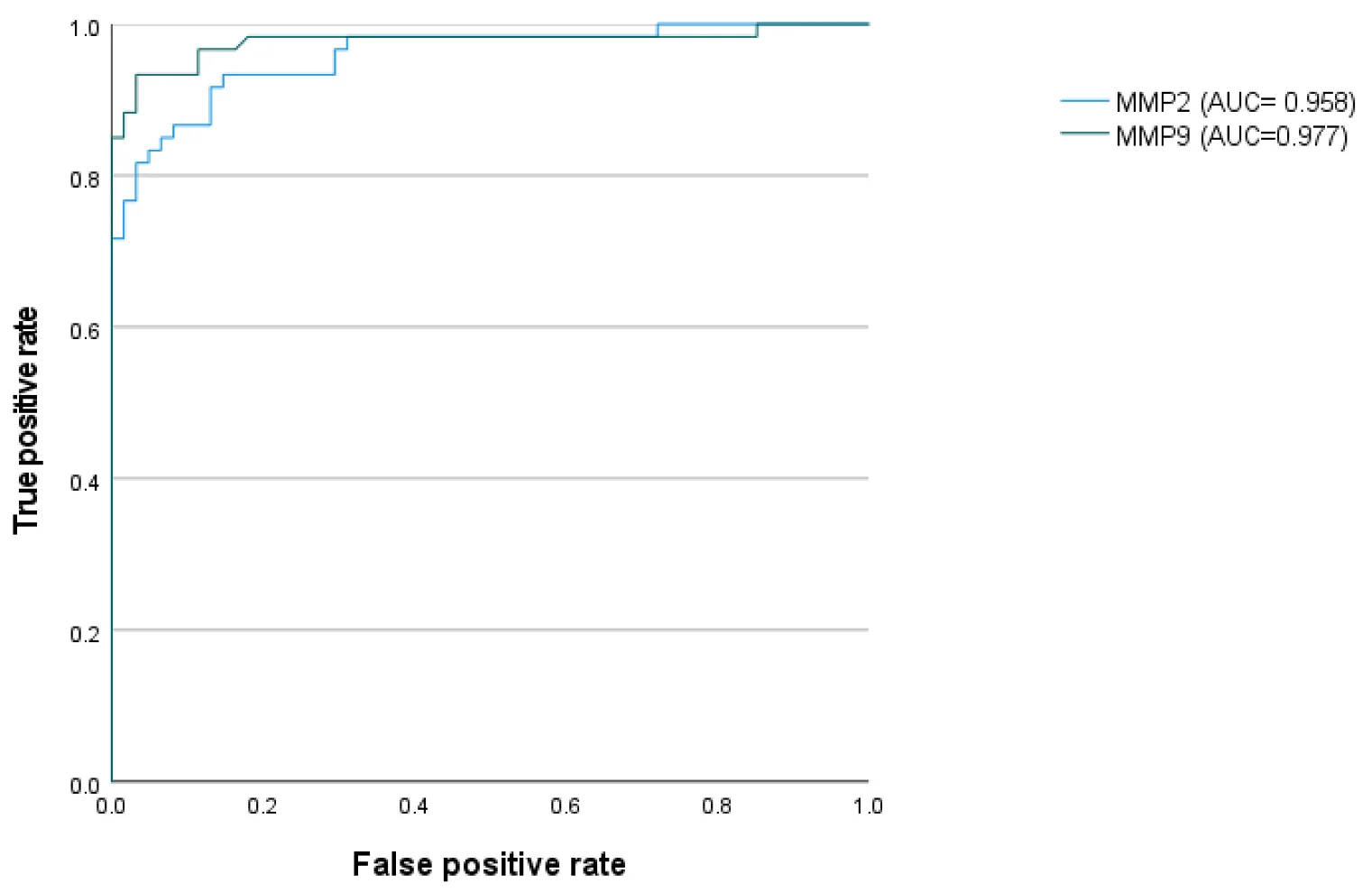
ROC curves demonstrating strong discriminatory performance of tissue MMP2 and MMP9.

When each tissue marker was modelled with adjustment for age, BMI, and parity, the values of COL1, COL3, ELN, MMP2, MMP3, and MMP9 remained independently associated with POP/SUI group (Table 4 and Figure 2). The largest adjusted effect sizes were observed for MMP9 (adjusted OR per SD 184.29, 95% CI 13.40–2534.30, *p* < 0.001) and MMP2 (adjusted OR per SD 30.79, 95% CI 6.75–140.43, *p* < 0.001). COL1 and ELN showed protective inverse associations after adjustment, while MMP1 lost independent significance (adjusted *p* = 0.506).

### 3.5. Combined Model Performance

The highest apparent discriminatory performance was observed for the three-marker tissue model (COL3, MMP2, and MMP9; apparent AUC 0.998). Under stratified 10-fold cross-validation, the fold-wise mean AUC ± SD was 0.978 ± 0.049 for the two-marker tissue model, 0.986 ± 0.035 for the three-marker tissue model, 0.989 ± 0.027 for the five-marker tissue model, and 0.687 ± 0.219 for the three-marker plasma model. Using pooled out-of-fold predictions, the three-marker tissue model achieved an AUC of 0.984 (95% CI 0.960–1.000), sensitivity 0.950 (95% CI 0.887–1.000), specificity 0.984 (95% CI 0.944–1.000), PPV 0.983 (95% CI 0.942–1.000), and NPV 0.952 (95% CI 0.894–1.000). The two-marker tissue model showed similarly strong internally validated performance, whereas the five-marker model did not provide a consistent advantage over the more parsimonious three-marker panel. In contrast, the three-marker plasma model yielded a pooled out-of-fold AUC of 0.690 (95% CI 0.592–0.783), with sensitivity 0.633 (95% CI 0.508–0.754) and specificity 0.705 (95% CI 0.586–0.815) (Table 5).

### 3.6. Correlation of Biomarkers with Clinical Severity

Within the POP group, MMP9 showed moderate positive correlations with several anatomical POP-Q measurements (Bp, Ba, Aa, and C; rho = 0.516–0.577, all *p* < 0.001). MMP2 was positively correlated with POP-Q C (rho = 0.364, *p* = 0.004), whereas its correlation with PFDI-20 was not statistically significant. MMP9 was not significantly correlated with PFDI-20 or UDI-6, and COL1 showed a moderate inverse correlation with POP-Q Bp (rho = −0.386, *p* = 0.002) (Table 6).

### 3.7. Tissue Morphological Changes

**Figure 3:**
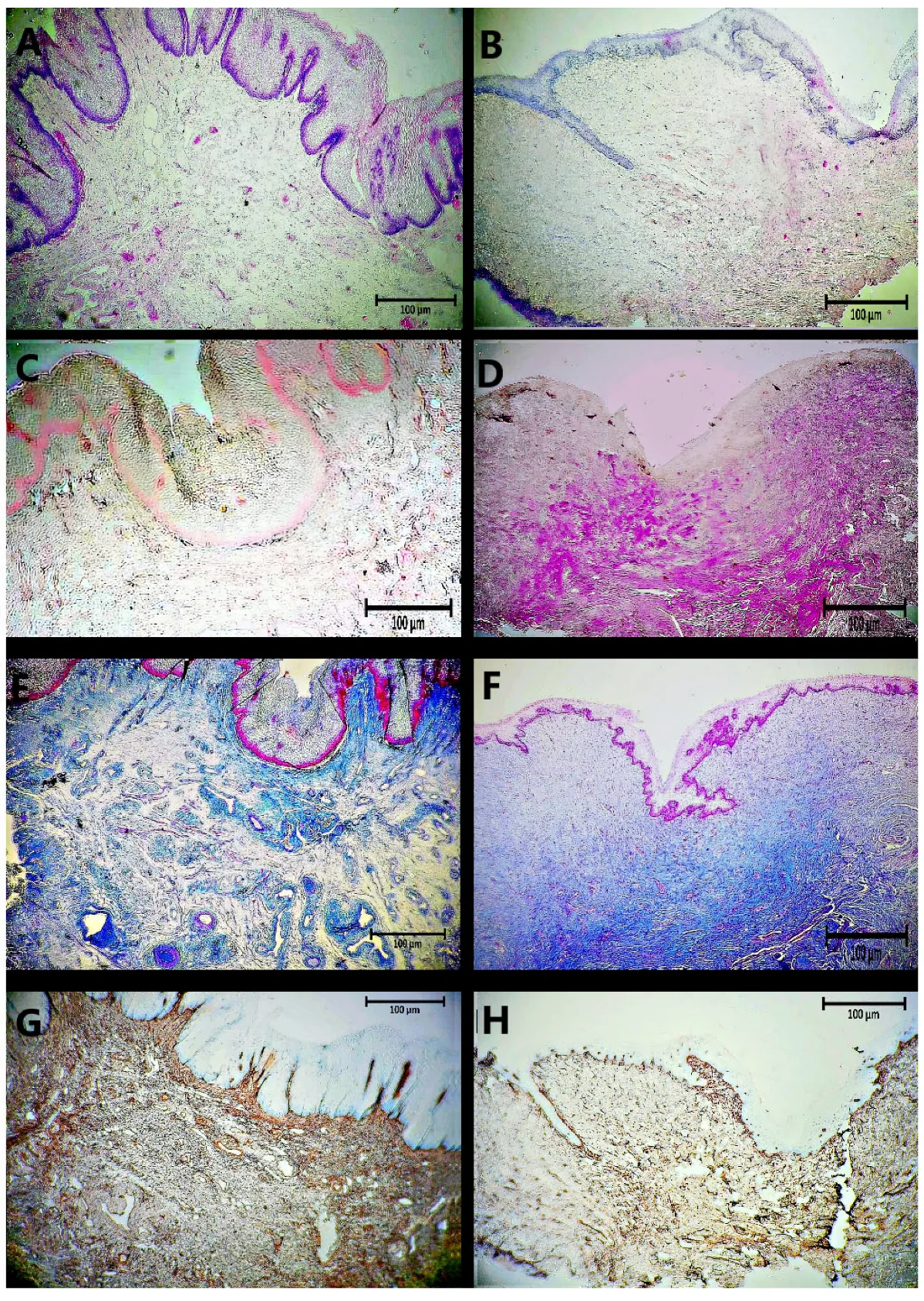
Histopathological and immunohistochemical characteristics of vaginal-wall connective tissue in control and POP/SUI groups (magnification ×40, scale bare 100 μm). Representative photomicrographs showing tissue morphology, collagen distribution, and vimentin expression. (**A**,**B**) Hematoxylin and eosin (H&E) staining—the control group (**A**) demonstrates a denser connective tissue architecture with thicker collagen bundles and lower apparent cellularity, whereas the POP/SUI group (**B**) exhibits a looser stromal organisation with increased apparent cellularity and less conspicuous collagen fibres. (**C**,**D**) Van Gieson staining—the control group (**C**) shows dense collagen deposition with prominent red-stained collagen bundles, while the POP/SUI group (**D**) demonstrates a more diffuse and less compact collagen distribution. (**E**,**F**) Masson’s Trichrome staining—extensive blue-stained collagen fibres are observed in the control group (**E**), indicating a greater extracellular matrix content, whereas the POP/SUI group (**F**) shows thinner and more loosely arranged collagen bundles. (**G**,**H**) Vimentin immunohistochemistry—strong and diffuse vimentin immunoreactivity is present in the control group (**G**), while the POP/SUI group (**H**) demonstrates qualitatively weaker and less extensive vimentin immunoreactivity.

In the control group, the connective tissue appears denser and more collagen-rich, characterised by thicker, more organised collagen fibres and reduced apparent cellularity (Figure 3A). The connective tissue stained with VG demonstrated a denser stromal architecture with more prominent red-stained collagen fibre bundles. Collagen deposition appeared increased, particularly within the central and deeper connective tissue layers, resulting in a more compact connective tissue structure (Figure 3C). The connective tissue stained with Masson’s Trichrome exhibited more extensive blue staining of collagen fibres, indicating a greater amount of connective tissue matrices. The collagen bundles appeared thicker, denser, and more widely distributed throughout the stroma (Figure 3E). The control group exhibited strong and diffuse vimentin immunoreactivity within the connective tissue stroma, indicating abundant vimentin-positive mesenchymal cells (Figure 3G).

In the POP/SUI group, the connective tissue exhibits a relatively loose stromal architecture with greater apparent cellularity and less conspicuous collagen bundles (Figure 3B). In the VG-stained sections, collagen fibres appeared as bright-red, while the remaining connective tissue components and cytoplasm appeared yellow, demonstrating an even distribution of collagen throughout the stroma without evidence of excessive extracellular matrix accumulation (Figure 3D). Masson’s Trichrome staining revealed moderate collagen distribution within the connective tissue. Collagen fibres stained blue, while epithelial and cytoplasmic components stained red to pink. The collagen bundles appeared relatively thin and loosely arranged (Figure 3F). The experimental group showed weaker and less extensive vimentin immunoreactivity compared with the control group (Figure 3H).

## 4. Discussion

The principal finding of the present study is that the tissue-level ECM remodelling represents the strongest and most coherent biological signal associated with pelvic floor dysfunction. Compared with controls, affected women had lower tissue COL1 and ELN (structural integrity markers), higher COL3, and significantly higher MMP2 and MMP9 protein concentrations. The consistency of these findings strongly exceeded the results of the plasma parameters. The two-marker model based on MMP2 and MMP9 results (Table 5) indicates that these tissue MMP concentrations capture a substantial portion of the case–control biomarker. On the other hand, the three-marker plasma model, which shows significantly lower performance, supports the interpretation that local tissue biomarkers provide substantially greater discriminatory value than systemic measurements in this cohort. Given the case–control design and the clear biological separation between groups, these high AUC values should still be interpreted with caution. The results of the pathohistological analysis show significant alterations in ECM structure (e.g., fibre abundance and distribution) in the POP/SUI group (Figure 3). The observed pattern aligns with a localised ECM-remodelling phenotype in clinically relevant prolapse; because no isolated SUI group was studied, these tissue findings cannot be attributed independently to SUI [4,9,10,11,12,13,14,15].

Tissue COL1 was lower in the POP/SUI group by approximately 12.45 ng/mg (33.67 ± 9.05 vs. 46.12 ± 7.36, *p* < 0.001, Table 3). Because collagen I is the primary load-bearing collagen in pelvic support tissues, this reduction is biologically consistent with impaired tensile integrity. The direction of effect is concordant with early biochemical work showing altered collagen metabolism in genitourinary prolapse and with later tissue studies demonstrating reduced collagen I expression in uterosacral ligaments and prolapsed vaginal tissues [6,7,16]. The recent meta-analysis also supports lower collagen I as one of the most reproducible ECM abnormalities across studies [9]. In our cohort, the signal remained independent after adjustment, arguing that the COL1 deficit is not explained solely by age, BMI, or parity.

COL3 was significantly higher in the POP/SUI group (41.11 ± 6.00 vs. 33.23 ± 5.78, *p* < 0.001, Table 3). A relative increase in type III collagen is commonly interpreted as a remodelling or repair that is mechanically inferior to a collagen I-dominant architecture. This interpretation is consistent with the prior prolapse and stress urinary incontinence literature showing altered collagen subtype balance in vaginal connective tissue and with recent reviews emphasising that the collagen I/III disequilibrium is a recurring feature of POP biology [7,9,10,11]. In the adjusted model, COL3 remained independently associated with POP/SUI group status, supporting the concept that our findings reflect active matrix remodelling rather than only demographic confounding.

ELN was substantially lower in the POP/SUI group, and its depletion reflects pelvic support since the tissues must provide recoil as well as tensile resistance. Reduced elastin content and abnormal elastic fibre organisation have been reported previously in prolapsed tissues, and the recent meta-analytic literature confirms that elastin reduction is among the most consistent structural findings in POP and SUI [8,9,12,17]. Our data extend that evidence by showing that tissue elastin not only differs between groups, but also retains an independent inverse association with case status after adjustment.

Finally, the changes in MMP2 and MMP9 were found to be the most powerful markers in our cohort (Table 5 and Table 6, Figure 2), which aligns with the literature survey. Earlier studies demonstrated increased MMP-2 expression and altered TIMP balance (not measured in the present study) in the uterosacral ligaments, as well as increased MMP-1 and MMP-9 in the uterosacral and vaginal tissues of women with prolapse [18,19]. More recent evidence has reinforced an MMP-associated ECM-remodelling model, and a meta-analysis identified higher MMP-1, MMP-2, and MMP-9 expression across pooled studies [9]. Some previous studies on tissue again reported significantly increased immunoreactivity for MMP-1, MMP-2, and MMP-9 in prolapsed ligaments [20]. Our adjusted models strengthen these interpretations, with MMP2 showing an adjusted OR per SD of 30.79 (95% CI 6.75–140.43, *p* < 0.001) and MMP9 an adjusted OR per SD of 184.29 (95% CI 13.40–2534.30, *p* < 0.001). The two-marker and three-marker tissue models both achieved near-perfect AUC values, demonstrating a strong association between increased tissue MMP levels and the POP-associated tissue phenotype. Because the ELISA assays quantified MMP protein concentrations rather than enzymatic activity, these findings should not be interpreted as direct evidence of increased net proteolytic activity.

MMP1 and MMP3 were also found to be higher in prolapse tissue on univariate comparison. However, after adjustment, MMP1 lost statistical significance (adjusted *p* = 0.506), whereas MMP3 remained independently associated with POP (Table 4). One can speculate that there is broader ECM dysregulation in POP, with multiple MMP family members affected, and that the most reproducible signals across studies remain MMP-2 and MMP-9. On the other hand, MMP-1 often behaved as a contextual marker of remodelling intensity rather than a uniformly independent determinant [9,19,20]. These explanations fit into the obtained results.

At the plasma level, only COL1 (*p* = 0.007 and δ = −0.283) and ELN (*p* < 0.001 and δ = −0.438) were significantly different between the groups (Table 2), and the best plasma model performed significantly worse than the best tissue models. It should be noted that EDTA plasma samples were used in this study, whereas a substantial proportion of previous studies have reported serum concentrations of ECM-related biomarkers. Because the coagulation process can induce platelet and leukocyte activation and release of matrix metalloproteinases (particularly MMP-9), serum measurements may be artificially elevated compared to plasma. This methodological difference should be considered when interpreting absolute biomarker levels and may partly explain variability across studies. Nonetheless, the results obtained support the interpretation that local tissue remodelling is much closer to the disease process than systemic spillover measurements. Recent reviews frame POP primarily as a localised connective tissue disorder, in which the most important molecular changes are detected in the affected ligaments and vaginal wall rather than in the peripheral circulation [10,11,21]. This was also supported by changes in the expression and position of collagen fibres in the samples examined (Figure 3).

Taken together, our results identify a POP-associated vaginal-wall ECM phenotype characterised by lower structural matrix proteins (COL1 and elastin), relative enrichment of remodelling-associated COL3, and higher MMP2 and MMP9 protein concentrations. Within the POP group, MMP9 retained moderate correlations with several anatomical POP-Q measurements, whereas several of the very high full-cohort correlations attenuated when case–control separation was removed. These observations support an association with the POP tissue phenotype but do not establish a causal mechanism. This interpretation is consistent with recent studies linking ECM disorganisation, mechano-transduction abnormalities, transcriptomic dysregulation, and altered fibroblast signalling to the progression of POP [4,9,12,13,14,15]. Recent studies have also highlighted pathway-level targets linked to ECM turnover, fibroblast signalling, and molecular risk architecture, and emerging computational work is beginning to identify novel ECM biomarker candidates for POP [13,14,15,21,22,23]. Within that landscape, the prominence of tissue MMP2 and MMP9 in our cohort identifies them as candidate tissue biomarkers for future validation. Due to the fact that SUI occurred only concomitantly with POP and no isolated SUI group was studied, the present findings cannot characterise an independent SUI-associated ECM phenotype.

Strengths of this analysis include the internally consistent case–control design, standardised tissue sampling from the same anatomical site, simultaneous evaluation of plasma and tissue biomarkers, and explicit internal resampling of candidate models. The main limitations are the moderate sample size, baseline differences between groups, single-centre design, and the fact that the biological signal is driven mainly by clinically significant prolapse rather than isolated SUI. The near-perfect apparent discrimination of the tissue models should therefore be interpreted in the context of biologically distinct case–control groups. The candidate panels were selected after examination of the full-cohort univariate and adjusted results and only then fixed for resampling; thus, cross-validation does not account for the preceding data-informed panel-selection step. Cross-validation reduced optimism and demonstrated internal stability, but it does not substitute for testing in an independent cohort. External validation was not available for the present study and is required before any clinical diagnostic or prognostic application. In addition, PPV and NPV are prevalence-dependent and, in this approximately balanced case–control sample, should be interpreted only as cohort-specific classification measures. Wet-tissue-weight normalisation may also be influenced by variation in tissue hydration. From a translational perspective, the present findings support a tissue-centred biological framework rather than a ready-to-use diagnostic test. In addition, TIMP levels were not quantified in the present study. As MMP-mediated extracellular matrix turnover is regulated by the balance between MMPs and their endogenous tissue inhibitors, simultaneous assessment of TIMPs would provide additional context for interpreting MMP regulation within prolapsed tissue. Furthermore, menopausal status, hormonal therapy, and previous pelvic surgery were not included in the present analysis and therefore could not be accounted for as potential confounding factors.

## 5. Conclusions

The results of the present study show that pelvic organ prolapse is associated with consistent alterations in extracellular matrix composition at the tissue level that clearly exceed the discriminatory capacity of plasma biomarkers. The observed pattern of reduced collagen type I and elastin, increased collagen type III, and elevated matrix metalloproteinase levels, particularly MMP-2 and MMP-9, supports a biologically coherent model of structural weakening which is consistent with a biologically coherent pattern of structural matrix alteration accompanied by increased MMP protein concentrations. Tissue MMP-2 and MMP-9 emerged as the strongest individual discriminators of case status, and compact tissue-based models retained high discrimination during internal cross-validation. These estimates represent internal cohort discrimination and require external validation in an independent population before clinical use. Within the POP/SUI group, selected tissue biomarkers showed moderate associations with anatomical POP-Q measurements. In contrast, plasma biomarkers showed substantially weaker discrimination, suggesting that systemic measurements may not adequately capture the localised tissue-associated biomarker signal as clearly as vaginal-wall measurements in this cohort. Taken together, these observational findings demonstrate an association between pelvic organ prolapse and a localised vaginal-wall extracellular matrix remodelling phenotype; they do not establish that extracellular matrix dysfunction is the primary cause of POP or provide direct functional evidence of altered proteolytic activity. The findings provide a basis for future mechanistic studies and externally validated biomarker stratification studies.

## Figures and Tables

**Table 1:** Comparison of clinical characteristics between groups.

Examined Parameter	POP/SUI Group	Control	Statistical Parameters		
**Age (years)**	58.27 ± 8.24	50.95 ± 8.31	Student’s *t*-test	t = 4.863	<0.001
**BMI (kg/m^2^)**	29.60 ± 3.48	25.05 ± 3.18	Student’s *t*-test	t = 7.527	<0.001
**Parity**	3 (2–4)	2 (1–3)	Mann–Whitney U	U = 1167.5	<0.001
**Vaginal births**	2 (1–3)	1 (1–2)	Mann–Whitney U	U = 1181.5	<0.001
**Caesarean section**	0 (0–1)	0 (0–1)	Mann–Whitney U	U = 1827.5	0.988
**UDI-6 score**	31 (18–36)	0 (0–0)	Mann–Whitney U	U = 0.00	<0.001
**PFDI-20 score**	94 (73–110)	0 (0–0)	Mann–Whitney U	U = 0.00	<0.001

Values are presented as mean ± standard deviation (SD) for variables analysed using Student’s *t*-test, and as median (interquartile range) for variables analysed using the Mann–Whitney U test.

**Table 2:** Comparison of plasma biomarkers between groups.

Examined Parameter	POP/SUI Group	Control	Statistical Parameters		
**COL1 plasma (ng/mL)**	10.27 (6.98)	13.81 (6.47)	Mann–Whitney U	δ = −0.283	0.007
**COL3 plasma (ng/mL)**	1.28 (0.69)	1.43 (0.36)	Mann–Whitney U	δ = −0.195	0.064
**ELN plasma (ng/mL)**	2.49 (0.95)	3.20 (1.42)	Mann–Whitney U	δ = −0.438	<0.001
**MMP1 plasma (ng/mL)**	0.96 (1.13)	0.85 (0.69)	Mann–Whitney U	δ = 0.084	0.423
**MMP2 plasma (ng/mL)**	1.90 (6.66)	2.86 (20.43)	Mann–Whitney U	δ = −0.124	0.238
**MMP3 plasma (ng/mL)**	5.03 ± 2.21	5.46 ± 1.64	Student’s *t*-test	d = −0.221	0.227
**MMP9 plasma (ng/mL)**	2.87 (3.96)	3.46 (3.42)	Mann–Whitney U	δ = −0.106	0.316

Values are presented as mean ± standard deviation (SD) for variables analysed using Student’s *t*-test, and as median (interquartile range) for variables analysed using the Mann–Whitney U test. Effect sizes are reported as Cohen’s d for Student’s *t*-test and Cliff’s delta (δ) for Mann–Whitney comparisons; positive values indicate higher values in the POP/SUI group and negative values indicate lower values.

**Table 3:** Comparison of tissue biomarkers between groups and their discriminatory performance.

Examined Parameter	POP/SUI Group	Control	Statistical Parameters				
**COL1 (ng/mg)**	33.67 (9.05)	46.12 (7.36)	Mann–Whitney U	δ = −0.795	<0.001	0.898	<0.001
**COL3 (ng/mg)**	41.11 ± 6.00	33.23 ± 5.78	Student’s *t*-test	d = 1.340	<0.001	0.818	<0.001
**ELN (ng/mg)**	21.63 ± 4.24	27.57 ± 3.92	Student’s *t*-test	d = −1.457	<0.001	0.846	<0.001
**MMP1 (ng/mg)**	5.61 ± 1.16	4.53 ± 1.17	Student’s *t*-test	d = 0.877	<0.001	0.751	<0.001
**MMP2 (ng/mg)**	132.44 (22.96)	98.72 (19.63)	Mann–Whitney U	δ = 0.916	<0.001	0.958	<0.001
**MMP3 (ng/mg)**	17.45 ± 2.95	14.83 ± 2.79	Student’s *t*-test	d = 0.912	<0.001	0.743	<0.001
**MMP9 (ng/mg)**	216.63 (40.91)	145.02 (27.80)	Mann–Whitney U	δ = 0.954	<0.001	0.977	<0.001

Values are presented as mean ± standard deviation (SD) for variables analysed using Student’s *t*-test, and as median (interquartile range) for variables analysed using the Mann–Whitney U test. Effect sizes are reported as Cohen’s d for Student’s *t*-test and Cliff’s delta (δ) for Mann–Whitney comparisons; positive values indicate higher values in the POP/SUI group and negative values indicate lower values.

**Table 4:** Adjusted logistic models for tissue biomarkers.

Marker	Adjusted OR per 1 SD (95% CI)	Adjusted *p*	Univariate AUC	Adjusted Model AUC
**COL1 (ng/mg)**	0.10 (0.03–0.29)	<0.001	0.898	0.963
**COL3 (ng/mg)**	4.05 (1.82–8.99)	<0.001	0.818	0.930
**ELN (ng/mg)**	0.18 (0.08–0.41)	<0.001	0.846	0.946
**MMP1 (ng/mg)**	1.24 (0.65–2.36)	0.506	0.751	0.905
**MMP2 (ng/mg)**	30.79 (6.75–140.43)	<0.001	0.958	0.980
**MMP3 (ng/mg)**	2.71 (1.44–5.09)	0.002	0.743	0.923
**MMP9 (ng/mg)**	184.29 (13.40–2534.30)	<0.001	0.977	0.988

**Table 5:** Apparent and internally cross-validated model performance.

Model	Apparent AUC	CV AUC (95% CI)	Sensitivity/ Specificity (95% CI)	PPV/NPV (95% CI)
Tissue two-marker (MMP2 + MMP9)	0.992	0.976 (0.937–1.000)	Sens: 0.933 (0.864–0.985) Spec: 0.984 (0.946–1.000)	PPV: 0.982 (0.942–1.000) NPV: 0.938 (0.873–0.986)
Tissue three-marker (COL3 + MMP2 + MMP9)	0.998	0.984 (0.960–1.000)	Sens: 0.950 (0.887–1.000) Spec: 0.984 (0.944–1.000)	PPV: 0.983 (0.942–1.000) NPV: 0.952 (0.894–1.000)
Tissue five-marker (COL1 + COL3 + ELN + MMP2 + MMP9)	0.999	0.968 (0.923–0.998)	Sens: 0.950 (0.887–1.000) Spec: 0.951 (0.891–1.000)	PPV: 0.950 (0.889–1.000) NPV: 0.951 (0.891–1.000)
Plasma three-marker (ELN + COL1 + MMP3)	0.719	0.690 (0.592–0.783)	Sens: 0.633 (0.508–0.754) Spec: 0.705 (0.586–0.815)	PPV: 0.679 (0.552–0.792) NPV: 0.662 (0.541–0.773)

Apparent AUC values were calculated from the full-cohort logistic models. Cross-validated (CV) metrics are based on pooled held-out predictions from stratified 10-fold cross-validation. The candidate panels had been selected after examination of the full-cohort univariate and adjusted results and were then fixed before resampling; consequently, the cross-validated estimates do not account for uncertainty introduced by this preceding data-informed panel-selection step. Classification within each held-out fold used a Youden-index threshold determined only from the corresponding training fold. The 95% confidence intervals were estimated by 5000 bootstrap resamples of held-out observations. Fold-wise mean AUC ± SD values were 0.978 ± 0.049, 0.986 ± 0.035, 0.989 ± 0.027, and 0.687 ± 0.219 for the tissue two-marker, tissue three-marker, tissue five-marker, and plasma three-marker models, respectively. PPV and NPV are cohort-specific because case prevalence was fixed by the case–control design and should not be interpreted as population predictive values.

**Table 6:** Exploratory Spearman correlations between selected tissue markers and clinical outcomes within the POP group (n = 60).

Tissue Marker	Outcome	Spearman Rho	*p*
MMP9 tissue (ng/mg)	POP-Q Bp	0.516	<0.001
MMP9 tissue (ng/mg)	POP-Q Ba	0.570	<0.001
MMP9 tissue (ng/mg)	POP-Q Aa	0.577	<0.001
MMP9 tissue (ng/mg)	POP-Q C	0.564	<0.001
MMP2 tissue (ng/mg)	PFDI-20 score	0.199	0.127
MMP2 tissue (ng/mg)	POP-Q C	0.364	0.004
MMP9 tissue (ng/mg)	PFDI-20 score	−0.134	0.307
MMP2 tissue (ng/mg)	POP-Q Ba	0.178	0.174
MMP2 tissue (ng/mg)	POP-Q Aa	0.176	0.180
MMP9 tissue (ng/mg)	UDI-6 score	−0.208	0.112
MMP2 tissue (ng/mg)	POP-Q Bp	0.247	0.057
COL1 tissue (ng/mg)	POP-Q Bp	−0.386	0.002

## Data Availability

The raw data supporting the conclusions of this article will be made available by the authors on request.

## Abbreviations

The following abbreviations are used in this manuscript:

AUC	Area Under the Curve
BMI	Body Mass Index
CI	Confidence Interval
COL1	Collagen Type I
COL3	Collagen Type III
DAB	3,3′-Diaminobenzidine
ECM	Extracellular Matrix
EDTA	Ethylenediaminetetraacetic Acid
ELISA	Enzyme-Linked Immunosorbent Assay
ELN	Elastin
H&E	Hematoxylin and Eosin
IQR	Interquartile Range
MMP	Matrix Metalloproteinase
MMP1	Matrix Metalloproteinase-1
MMP2	Matrix Metalloproteinase-2
MMP3	Matrix Metalloproteinase-3
MMP9	Matrix Metalloproteinase-9
MT	Masson’s Trichrome
OR	Odds Ratio
PBS	Phosphate-Buffered Saline
PFDI-20	Pelvic Floor Distress Inventory-20
POP	Pelvic Organ Prolapse
POP-Q	Pelvic Organ Prolapse Quantification System
ROC	Receiver Operating Characteristic
SD	Standard Deviation
SPSS	Statistical Package for the Social Sciences
SUI	Stress Urinary Incontinence
TGF-β	Transforming Growth Factor Beta
UDI-6	Urogenital Distress Inventory-6
VG	Van Gieson Stain
